# Ethical and Legal Issues in Interactive Health Communications: A Call for International Cooperation

**DOI:** 10.2196/jmir.2.1.e8

**Published:** 2000-03-31

**Authors:** Roberto J Rodrigues

Cyberspace is a fast-changing, globally-networked, multicultural, and multilingual information environment with vast possibilities [1-9]. It calls into question national and international borders, cultural and ethical standards, regulations, and laws, which it bypasses and challenges [10-13]. In the health sector, self-care, drugs sold over the Internet, and providing access to technical knowledge and alternative forms of healthcare to the general public have destabilized drug regulatory mechanisms and the traditional physician-patient relationship.

The Internet offers unprecedented power to provide users of healthcare information - patients, professionals, families, caregivers, educators, researchers, insurers, regulators, and policymakers - with data of unprecedented timeliness, accuracy, depth, and diversity. The very qualities that make the Internet such a rich marketplace of ideas - its decentralized structure, global reach, leveling of access to the tools of publication, immediacy of response, and ability to facilitate free-ranging interchange - also make it an exceptional channel for potential misinformation, unethical use, concealed bias, covert self-dealing, fraudulent practices, and evasion of legitimate regulation.

There are many inadequacies concerning national and international controls and legislation, especially regarding the issue of jurisdiction; and urgent need for an internationally accepted policy framework that addresses basic rights and responsibilities of users and providers. Freedom of access to information and expression and the protection of users' data security and privacy are especially critical topics. Decisions and initiatives related to cyberspace law and ethics issues in health and healthcare must necessarily involve experts from a variety of knowledge domains involving civil and criminal law, medical ethics (bioethics), computing ethics, medical computing, and legal medicine.

Given the sensitive nature of health care information, and the high degree of dependence of health professionals on reliable records, the issues of integrity, security, privacy, and confidentiality are of particular significance and must be clearly and effectively addressed by health and health-related organizations and professionals. Two factors make the matter a subject of preeminent significance: the intrinsically sensitive nature of patient data; and the growing use of network computing, particularly the Internet, for healthcare information processing. The growth of off-site processing and storage of electronic health records by application services providers (ASPs) adds a new dimension to those issues.

Maintaining and safeguarding the integrity and physical protection of data and systems, privacy and confidentiality of individual health information, quality of content, and the protection of consumers and online health industry commercial interests against unethical practices, are the areas of greatest concern in the implementation and use of Internet and other interactive applications in health and healthcare.

Privacy involves many aspects [14-18], and the issue has been consistently one of the top concerns of users. The emergence of health data banks has given rise to fears related to privacy, right of access, and intended use of personal data. In many countries, proposals and actual reform of the laws have been enacted, according to which individuals are entitled to know what information is stored about them, who accessed that information, and what mechanisms are available to correct erroneous information [16,19-22]. Authenticity, reliability, and accuracy of the health-related information available on the more than 20,000 health sites that are available are major issues. Many websites are profit-driven, others promote unproven and even dangerous forms of treatment or products, while others may be good intentioned but contain misleading or false information. To ascertain the credibility, motives, sponsorship, and eventual conflicts of interest of websites is an extremely difficult task [3-5,17,23-27]. In the center of this "free-for-all," physicians are increasingly confronted with Internet-savvy patients who come to the consultation with a heap of downloaded material, ready to discuss his self-diagnosed condition and the latest mainstream or alternative treatments [4,28].

Electronic transactions involve important regulatory and legal issues not yet fully addressed. Vigilance in the maintenance of legal and ethical standards in the advertising, promotion, and sale of medical products through the Internet is required. Those standards include: approval of products, devices, and drugs by regulatory agencies at the site where the purchaser resides; the determination where the transaction occurred - in the purchaser's or the vendor's jurisdiction; and which courts and law will govern any disputes [29-31].

Although there is a general agreement among conscientious professionals that all health websites should be held to the same standard and that the introduction of some form of "seal of approval" is an interesting proposition, enforcement is still a nebulous area. Particularly in the international setting there are complex issues of jurisdiction not yet addressed by laws or agreements.

A number of organizations, government agencies, and scientific publishers have been active in the establishment of standards and methods to measure and assure credibility of health websites. One of the first players is the Health on the Net Foundation, established in 1996 [32,33]. Another very active independent group more recently constituted, and congregating a large number of international stakeholders, is the Internet Healthcare Coalition [5,34,35]. Other groups include Internet [36-38] and scientific medical publishers [39], the American Medical Association, and European medical societies: for a discussion see [13]. Quality-assurance methodologies range from peer-review and professional authorship to open discussion in an open moderated or non-moderated forum and many approaches have been proposed in the evaluation, categorization, and labeling of health websites [24,40-47], the central issue being how to best protect the public interest.

Although security, privacy, and confidentiality are matters of concern in telecommunication-based clinical applications, and indeed major features of it, such applications raise new challenges regarding professional conduct and accountability, technical standards, licensure, and reimbursement [10,48]. Liability, model of care, and medical malpractice must be seen under a novel perspective as telemedicine involves more than one provider, usually geographically distant and subject to diverse practice and legal value systems.

The society and public authorities have the responsibility to make information considered as of "public good" universally available for educational, cultural, and social needs. The challenge is to define and implement concepts, such as public domain contents and universal access to networks and services; and to promote public welfare while encouraging private initiatives and protecting human dignity, personal rights, fair use, intellectual property rights, and rightful economic interests.

Traditionally, local standards are considered the yardstick against which health practice is evaluated, and they determine the parameters for eventual litigation. Remote conduction of health interventions brings forth, however, a whole new range of issues and ethical aspects in the telecare patient-provider relationship. Those issues have been reviewed and recommendations regarding a code of practice proposed [11,13]. Guidelines regarding the ethical and legal aspects of telemedicine are in the process of being developed by national and international trade, professional, and technical organizations and by national regulatory agencies. Medical software is increasingly considered as another form of a medical device. An extensive review of the legal aspects of telemedicine practice in the U.S. has recently been compiled in the Compendium of Telemedicine Laws [49].

Licensing and professional standards of care providers and regulation of e-commerce is done in many countries on a regional or state level. Validation of professional licensure, alternative and non-approved health practice, contending with fraudulent practice and misleading claims, regulation, and legal jurisdiction problems on a national and international basis are major regulatory and quality assurance problems in these circumstances.

The health sector has not addressed information security in a comprehensive manner. Healthcare organizations face a great variety of security, privacy, and confidentiality risks and must be made fully responsible for maintaining all aspects of security and confidentiality of data and information. Eventual conflicts between data sharing, data security, and confidentiality must be addressed early in the process of systems procurement and development and after implementation. Some health organizations have implemented security features in their information systems, but most organizations do not have written rules or procedures for their employees who are authorized to access client's information, such as policies on disclosure of sensitive information, or personnel policies dictating the types of disciplinary actions that will be taken if staff violate policies. Nevertheless, regulations and technical standards for privacy assurance and maintenance of data integrity and access security must be reasonable, in terms of recognizing the realities of health care delivery, the variability of application environments, and the diversity of national ethical values and legal systems.

Developing countries are particularly affected by the rapid expansion of interactive communication technologies - increasingly, governments, professional organizations, advocacy groups, and users in developing countries have expressed their concerns about the impact of such happenings. Particularly, there is great apprehension about the reliability of healthcare information, new forms of health practice, advertising and commercial processes, content appropriateness, and privacy, as they pertain to the Internet. Multilateral or international agencies and national technical cooperation agencies are promoting the deployment of IHC applications, but have mainly focused on technological infrastructure development; little has been done regarding contents, human resources, impact evaluation, and ethical and legal aspects [1,4,8,50-53].

Ethical and regulatory questions, and national and international legislation addressing the many issues related to quality of information in the Internet, e-commerce, and telemedicine are a matter of present concern of many international organizations. The United Nations, and particularly UNESCO, the International Telecommunications Union (ITU), the World Health Organization (WHO), the World Trade Organization (WTO), regional trade blocks (European Community, NAFTA, MERCOSUR), and multilateral agencies such as the World Bank and the Inter American Development Bank have been in the forefront of initiatives directed to the promotion of exchanges in this area.

Those are urgent and controversial issues that must be addressed jointly and comprehensively by international organizations, national and international scientific and technical societies, service providers, industry organizations, and users' interest groups, and not only from the viewpoint of legal or commercial interests. The United Nations specialized agencies, government organizations, independent and nonaligned consensus groups [5,32,36,54-56], and trustworthy service and content providers [29,36-38] are particularly well positioned to spearhead the discussions leading to the development of innovative policies for the area and the establishment of an ethical code of conduct focused on content, advertising and privacy issues, and fraud detection designed to ensure that consumers are provided with health information that is reliable and safe.

Roberto J. Rodrigues, Member, JMIR Editorial Board

Regional Advisor in Health Services Information Technology, Essential Drugs and Technology Program, Division of Health Systems and Services Development, Pan American Health Organization / World Health Organization, Washington, D.C., USA
